# Soluble Sema4D cleaved from osteoclast precursors by TACE suppresses osteoblastogenesis

**DOI:** 10.1111/jcmm.17416

**Published:** 2023-05-11

**Authors:** Takenobu Ishii, Montserrat Ruiz‐Torruella, Jae Young Kim, Hiroyuki Kanzaki, Abdullah Albassam, Wichaya Wisitrasameewong, Satoru Shindo, Roodelyne Pierrelus, Alireza Heidari, Umadevi Kandalam, Shin Nakamura, Alexandru Movila, Dmitriy Minond, Toshihisa Kawai

**Affiliations:** ^1^ Department of Orthodontics Tokyo Dental College Chiba Japan; ^2^ Institut Sant Joan Carrer de Sant Joan Barcelona Spain; ^3^ Department of Prosthodontics Yonsei University Dental Hospital Seoul Korea; ^4^ Department of orthodontics, School of Dental Medicine Tsurumi University Yokohama Japan; ^5^ Department of Endodontics, Faculty of Dentistry King Abdulaziz University Jeddah Saudi Arabia; ^6^ Department of Periodontology Faculty of Dentistry Chulalongkorn University Bangkok Thailand; ^7^ Department of Oral Science and Translational Research, College of Dental Medicine Nova Southeastern University Fort Lauderdale Florida USA; ^8^ Department of Pharmaceutical Sciences, College of Pharmacy Nova Southeastern University Fort Lauderdale Florida USA; ^9^ Center for Collaborative Research, Cell Therapy Institute Nova Southeastern University Fort Lauderdale Florida USA

**Keywords:** membrane type‐1 matrix metalloproteinase, osteoblastogenesis, osteoclasts, semaphorin 4D, sheddase, tumor necrosis factor alpha converting enzyme

## Abstract

Bone remodelling is mediated by orchestrated communication between osteoclasts and osteoblasts which, in part, is regulated by coupling and anti‐coupling factors. Amongst formally known anti‐coupling factors, Semaphorin 4D (Sema4D), produced by osteoclasts, plays a key role in downmodulating osteoblastogenesis. Sema4D is produced in both membrane‐bound and soluble forms; however, the mechanism responsible for producing sSema4D from osteoclasts is unknown. Sema4D, TACE and MT1‐MMP are all expressed on the surface of RANKL‐primed osteoclast precursors. However, only Sema4D and TACE were colocalized, not Sema4D and MT1‐MMP. When TACE and MT1‐MMP were either chemically inhibited or suppressed by siRNA, TACE was found to be more engaged in shedding Sema4D. Anti‐TACE‐mAb inhibited sSema4D release from osteoclast precursors by ~90%. Supernatant collected from osteoclast precursors (OC‐sup) suppressed osteoblastogenesis from MC3T3‐E1 cells, as measured by alkaline phosphatase activity, but OC‐sup harvested from the osteoclast precursors treated with anti‐TACE‐mAb restored osteoblastogenesis activity in a manner that compensates for diminished sSema4D. Finally, systemic administration of anti‐TACE‐mAb downregulated the generation of sSema4D in the mouse model of critical‐sized bone defect, whereas local injection of recombinant sSema4D to anti‐TACE‐mAb‐treated defect upregulated local osteoblastogenesis. Therefore, a novel pathway is proposed whereby TACE‐mediated shedding of Sema4D expressed on the osteoclast precursors generates functionally active sSema4D to suppress osteoblastogenesis.

## 1 INTRODUCTION

Semaphorins have been accepted as multipotent factors engaged in several physiological cellular events, such as neuronal growth regulation, immune regulation, epithelial remodelling and angiogenesis.^1^, ^2^ However, their functional roles in bone regeneration were only identified in 2011.^3^ Amongst the semaphorin family of some 20 sibling molecules, semaphorin 4D (Sema4D) was discovered to suppress the bone anabolic process.^3^ More specifically, Sema4D, produced by osteoclasts, downregulates IGF‐1‐mediated osteoblast activation by binding to plexin B1, a Sema4D receptor. In contrast, anti‐Sema4D‐mAb could regain lost bone contents in osteoporosis‐induced ovariectomized mice,^3^ suggesting the translational value of targeting Sema4D in bone lytic disorders.

Sema4D was initially identified as a type‐1 membrane protein expressed on immune cells,^4^ but its soluble form was also detected in plasma. It is also reported that the soluble form of Sema4D (sSema4D) is secreted by γδ T and cancer cells,^5^, ^6^ as well as platelets and osteoclasts.^7^ Since no studies have reported spliced form of Sema4D that can be secreted, it is plausible that detected sSema4D results from the cleavage of membrane‐bound Sema4D expressed on the cell surface. Important to this study, an elevated level of sSema4D was detected in the gingival crevice fluid (GCF) and serum of patients with inflammatory bone lytic diseases of periodontitis^8^ and rheumatoid arthritis,^9^ respectively. According to Li Zhu et al., the extracellular domain of Sema4D is shed from the platelet surface by the ectoenzyme TACE (TNF‐converting enzyme, also known as ADAM17).^7^ On the other hand, it was also reported that Sema4D expressed on the cellular membrane of cancer cells is shed by Membrane Type‐1 *Matrix Metalloproteinase* (*MT1*‐*MMP*) which has been implicated in cancer metastasis.^6^, ^10^ However, the mechanism responsible for producing sSema4D from osteoclasts is unknown. Therefore, this study aimed to identify the enzyme responsible for shedding Sema4D expressed on osteoclasts and evaluate the functionality of such sSema4D released from osteoclasts.

## 2 RESULTS AND DISCUSSION

### 2.1 Production of sSema4D by RANKL‐primed osteoclasts

**FIGURE 1:**
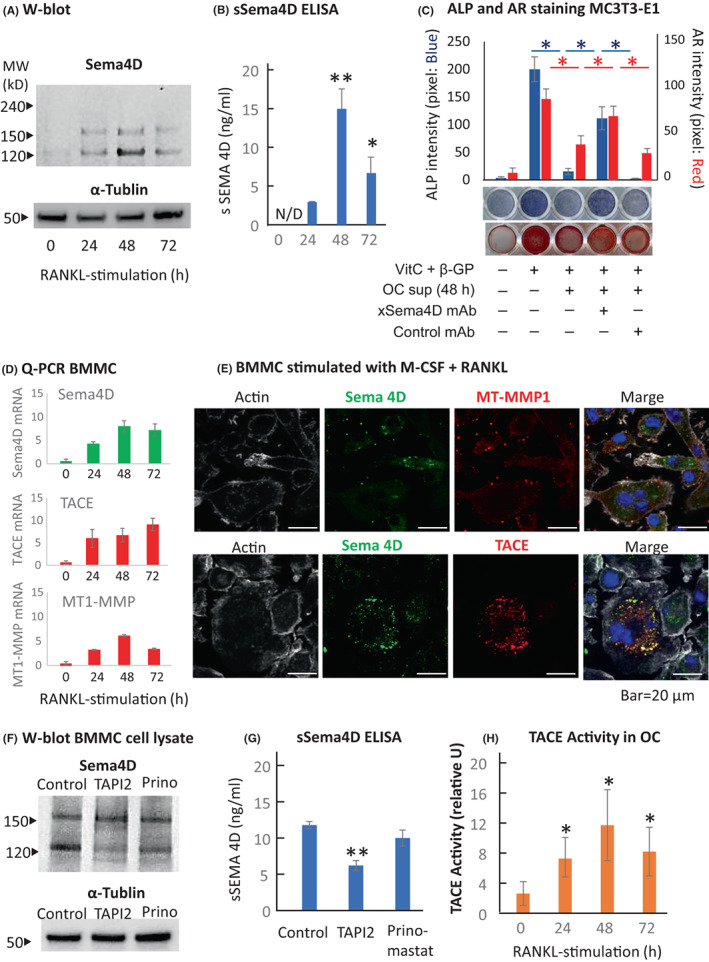
Sema4D is cleaved by osteoclast TACE to become a soluble form. (A) Upon stimulation of bone marrow mononuclear cells (BMMC: C57BL/6 mouse) with recombinant mouse RANKL (50 ng/ml, BioLegend), Sema4D was expressed on osteoclast cells in its membrane‐bound form (150 kD) and soluble form (120 kD) as determined by Western blot. (B) The level of soluble Sema4D showed a peak at 48 h after stimulation with RANKL, diminishing at 72 h. According to sSema4D ELISA (RayBiotech), the amounts of sSema4D detected in the culture supernatant of RANKL‐stimulated BMMCs (OC‐sup) were proportional to those detected in Western blot. (C) Osteoblastogenesis was induced by the stimulation of MC3T3‐E1 cells with ascorbic acid (Vit C) and β‐glycerophosphate (β‐GP) in the presence or absence of the OC‐sup harvested at 48 h with or without anti‐Sema4D‐mAb or control mAb. After 7‐day culture of MC3T3‐E1, the alkaline phosphatase (ALP) activity was detected to be reduced by the incubation with OC‐sup, which was abrogated by anti‐Sema4D‐mAb, but not by control mAb. For the detection of mineral deposition stained by Alizarin red (AR), MC‐3 T3‐E1 cultures were incubated for 3 weeks. Results showed a trend similar to that detected in APL staining. (D) Upon stimulation of BMMCs with RANKL, elevated expression of all three mRNAs for Sema4D, TACE and MT1‐MMP was detected by qPCR. (E) In a fluorescence confocal microscopy of osteoclasts stimulated by RANKL, colocalization of Sema4D and TACE was observed in the RANKL‐stimulated BMMCs, whereas no colocalization was detected between MT1‐MMP and Sema4D. (F) The production of sSema4D from RANKL‐stimulated BMMCs was prominently suppressed by TAPI2, an inhibitor of ADAM 8, 10, 12 and TACE, whereas Prinomastat, an inhibitor of MMP‐2, MMP‐9 and MT1‐MMP, showed a reduced capacity to suppress the production of sSema4D, as measured by Western blot (120KD band). (G) The ELISA measurements of TAPI2 and Prinomastat sSema4D in RANKL‐stimulated BMMC showed that TAPI2 significantly inhibited sSEMA4D. *p* < 0.01. (H) The sheddase activity of TACE expressed on RANKL‐stimulated BMMCs was confirmed by InnoZyme™ TACE Activity Kit (Sigma‐Aldrich). *p < 0.05 and **p < 0.01, by one‐way anova, followed by Tukey's post hoc test

Upon stimulation of bone marrow mononuclear cells (BMMC: C57BL/6 mouse) with recombinant mouse M‐CSF and RANKL (20 ng/ml and 50 ng/ml, respectively, both from BioLegend), Sema4D was expressed on osteoclast precursors in its membrane‐bound form (150 kD) and soluble form (120 kD) in W‐blot (Figure 1A). The release of sSema4D in the culture supernatant of RANKL‐stimulated BMMCs (osteoclast [OC]‐sup) showed its highest level at 48 h, followed by a decline at 72 h. Importantly, the release of sSema4D from RANKL‐stimulated BMMCs occurred during the early stage of osteoclast differentiation (up to 72 h) before the start of cell–cell fusion. The number of nuclei detected in the cells was, at most, three. According to sSema4D ELISA (RayBiotech), the amounts of sSema4D in the OC‐sup were proportional to those detected in W‐blot (Figure 1B). Next, osteoblastogenesis was induced by the stimulation of MC3T3‐E1 cells with ascorbic acid (VitC) and β‐glycerophosphate (β‐GP) in the presence or absence of OC‐sup harvested at 48 h with or without anti‐Sema4D‐mAb^11^ or control mAb. Osteoblastogenesis activity, as monitored by alkaline phosphatase (ALP) staining and mineral deposition detected by Alizarin red (AR), showed that the OC‐sup suppressed osteoblastogenesis, whereas such suppression was abrogated by anti‐Sema4D‐mAb, but not control‐mAb. These results indicated that RANKL‐stimulated BMMCs can release sSema4D that is functionally active in suppressing osteoblastogenesis, corroborating the reports by others.^3^, ^12^

### 2.2 Cellular localization and sheddase activity by TACE and MT1‐MMP expressed by RANKL‐primed osteoclasts

To explain the generation of sSema4D from its membrane‐bound form, two ectoenzymes that are TACE and MT1‐MMP have been reported to shed membrane‐bound Sema4D.^6^, ^7^ Upon stimulation of BMMCs with RANKL, elevated expression of all three mRNAs for Sema4D, TACE and MT1‐MMP was detected by qPCR (Figure 1D). According to fluorescence confocal microscopy, colocalization of Sema4D and TACE was observed in the RANKL‐stimulated BMMCs but not the colocalization of MT1‐MMP and Sema4D (Figure 1E). The production of sSema4D from RANKL‐stimulated BMMCs was prominently suppressed by TAPI2, an inhibitor of ADAM 8, 10, 12 and TACE, with Ki values of 10, 3, 100 and 0.12 μM, respectively,^13^ as measured by Western blot (Figure 1F: 120 KD band) and ELISA (Figure 1G). Prinomastat, an inhibitor of MMP‐2, MMP‐9 and MT1‐MMP, with Ki values at 50, 150 and 300 pM, respectively,^14^ showed a lesser capacity to suppress the production of sSema4D (Figure 1F,G). The sheddase activity of TACE was elevated in the RANKL‐stimulated BMMCs stimulated with RANKL which peaked at 48 h (Figure 1H). These results suggested that TACE, but not MT1‐MMP, may be engaged in the shedding of membrane‐bound Sema4D expressed on RANKL‐stimulated BMMCs.

### 2.3 siRNA‐based loss‐of‐function assay to evaluate the role of the sheddases TACE and MT1‐MMP in generating sSema4D

**FIGURE 2:**
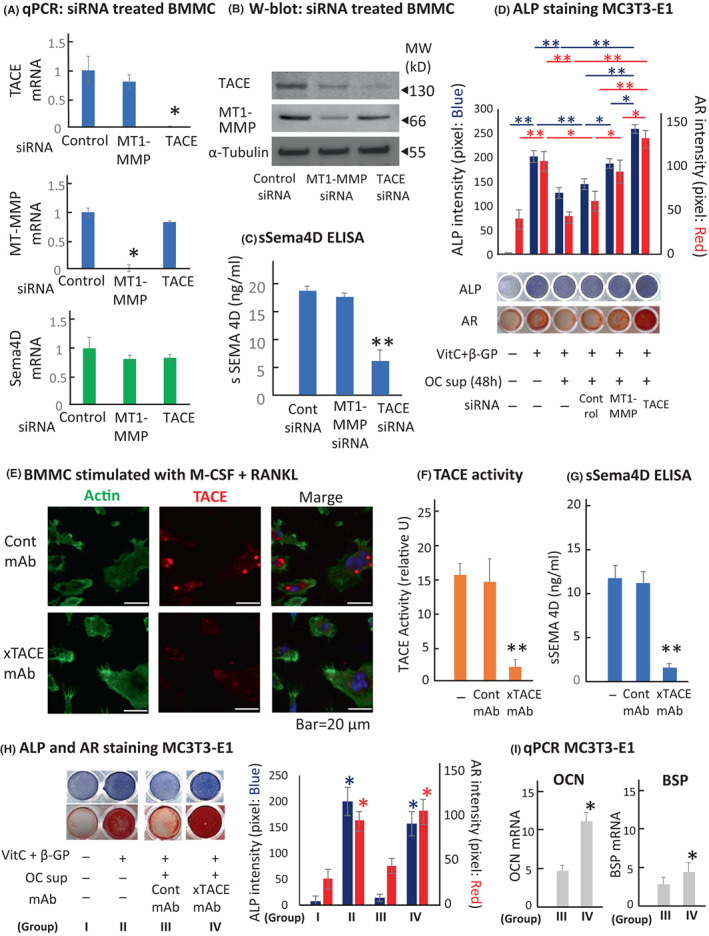
Osteoclast TACE regulates osteoblast differentiation by cleaving Sema4D. The treatment of RANKL‐stimulated BMMCs with anti‐TACE‐siRNA or anti‐MT1‐MMP‐siRNA suppressed the mRNA expressions for TACE and MT1‐MMP (24 h). (B) The protein expression levels of the above‐noted BMMCs were monitored by Western blotting (48 h). Anti‐MT‐MMP1‐siRNA and anti‐TACE‐siRNA treatment suppressed the expression of MT‐MMP1 and TACE. (C) Quantification of sSema4D using ELISA showed that the release of sSema4D from RANKL‐stimulated BMMC was inhibited by anti‐TACE‐siRNA, but not by anti‐MT‐MMP1‐siRNA (48 h). (D) The osteoblastogenesis induced in the MC‐3 T3‐E1 cells by in vitro stimulation with or without VitC and β‐GP for 7 days was monitored by ALP staining (Group II and Group I, respectively). OC‐sup from RANKL‐stimulated BMMCs suppressed the ALP expression in the VitC and β‐GP‐stimulated MC3T3‐E1 cells (Group III) which was abrogated by the addition of anti‐TACE‐siRNA treated OC‐sup (Group VI), but not that of control‐siRNA (Group IV) or anti‐MT1‐MMP‐siRNA (Group V). (E) The incubation of RANKL‐stimulated BMMCs with anti‐TACE‐mAb diminished the expression of TACE on the surface of osteoclast precursors, compared to that incubated with control mAb, as monitored by fluorescence confocal microscopy (48 h) (Reagents used: FITC‐conjugated phalloidin, rabbit anti‐TACE Ab, followed by Alexa 645‐anti‐rabbit IgG, DAPI). (F) Anti‐TACE‐mAb suppressed TACE activity detected in the OC‐sup (48 h). (G) Incubation of RANKL‐stimulated BMMC with anti‐TACE‐mAb, but not control mAb, reduced the release of soluble Sema4D. (H) Osteoblastogenesis detected by ALP and AR staining of the MC3T3‐E1 stimulated with VitC and β‐GP for 7 days and 21 days, respectively, (Group II; VitC + β‐GP, Group I; control no stimulation) was reduced by the addition of OC‐sup containing control mAb (Group III) was abrogated by the addition of anti‐TACE‐mAb (Group IV). (I) The MC3T3‐E1 cells collected from H‐Group III and H‐Group IV at 24 h were subject to qPCR for osteocalcin (OCN) mRNA and bone sialoprotein (BSP) mRNA expressions. Both osteoblastogenesis‐associated genes were significantly upregulated by the addition of anti‐TACE‐mAb. *p < 0.05 and **p < 0.01, by one‐way anova, followed by Tukey's post hoc test

Since two chemical inhibitors used for inactivation of TACE (Figure 1F,G) also target other enzymes, the RNAi‐based specific gene silence method was employed. The treatment of RANKL‐stimulated BMMCs with anti‐TACE‐siRNA or anti‐MT1‐MMP‐siRNA suppressed both mRNA and protein for TACE and MT1‐MMP (Figure 2A). As expected, the anti‐TACE‐siRNA, but not anti‐MT1‐MMP‐siRNA, suppressed the generation of sSema4D in the OC‐sup (Figure 2C). Anti‐MT1‐MMP‐siRNA also modestly suppressed the expression of TACE (Figure 2B), but the level of sSema4D production by RANKL‐stimulated BMMCs was still not significantly affected by it (Figure 2C). Osteoblastogenesis in MC3T3‐E1 cells was induced by stimulation with VitC and β‐GP. When the OC‐sup (Group‐III) was added to VitC/β‐GP‐stimulated MC3T3‐E1 cells, ALP expression was suppressed. However, treatment of RANKL‐primed BMMC with anti‐TACE‐siRNA (Group‐VI), but not anti‐MT1‐MMP siRNA (Group‐V), restored ALP expression, as well as Alizarin‐red (AR)‐stained mineral deposition, in MC3T3‐E1 cells from the level suppressed by OC‐sup (Group‐III) (Figure 2D). These results from our siRNA‐based loss‐of‐function assay confirmed that the release of sSema4D from RANKL‐stimulated BMMCs may be mediated by TACE, but not MT1‐MMP, corresponded to the results shown in Figure 1, which demonstrated that profound TACE inhibitor, TAPI2, prevented the shedding of membrane‐bound Sema4D expressed on RANKL‐stimulated BMMCs, implicating that TACE may be responsible for the shedding of Sema4D from osteoclast precursors.

### 2.4 Effects of anti‐TACE‐mAb on sSema4D release from RANKL‐stimulated BMMCs

We have demonstrated that TACE can shed the membrane‐RANKL expressed on T cells and B cells using a polyclonal rabbit anti‐human TACE neutralizing antibody.^15^ In the present study, anti‐mouse TACE‐neutralizing‐mAb (anti‐TACE‐mAb, mouse IgG1) was generated by immunizing a peptide antigen specific to mouse TACE (NTCKLLVVADHRFYKMG). The incubation of RANKL‐stimulated BMMCs with anti‐TACE‐mAb diminished the expression of TACE on the surface of osteoclast precursors, compared to that incubated with control‐mAb, as monitored by fluorescence confocal microscopy (Figure 2E). Anti‐TACE‐mAb also significantly suppressed TACE activity, as well as sSema4D in the OC‐sup (Figure 2F,G). Finally, to determine the effects of anti‐TACE‐mAb on the anti‐osteoblastogenesis activity elicited by the OC‐sup, ALP and AR staining was performed on MC3T3‐E1 cells stimulated with VitC and βGP in the presence or absence of OC‐sup and/or mAb (Figure 2H). Whilst osteoblastogenesis was suppressed by the OC‐sup incubated with control‐mAb, the OC‐sup incubated with anti‐TACE‐mAb completely rescued osteoblastogenesis (Figure 2H). Significantly elevated levels of mRNAs for osteocalcin (OCN) and bone sialoprotein (BSP) were observed in MC3T3‐E1 cells that received the OC‐sup incubated with anti‐TACE‐mAb, compared to that of control‐mAb (Figure 2I), suggesting that the osteoblastogenesis activity suppressed by the OC‐sup had been restored by anti‐TACE‐mAb. Taken collectively, this evidence suggested that anti‐TACE‐mAb‐mediated neutralization of sheddase activity of TACE expressed on osteoclast precursors can inhibit the release of uncoupling factor Sema4D from osteoclast precursors, which should promote the bone regeneration in the physiological context.

### 2.5 Possible role of TACE and sSema4D in regulating osteoblastogenesis in a mouse model with critical‐sized bone defect

We assessed the possible role of TACE using a critical‐sized bone defect (2 mm Φ) created in the calvaria bone of mice^16^ by administration with or without anti‐TACE‐mAb (i.p.). To examine the effect of sSema4D on local osteoblastogenesis, recombinant sSema4D was injected to the bone defect site administered with anti‐TACE‐mAb. According to the in vivo fluorescence imaging system, mineral deposition activity (RFU of OsteoSense 680EX) by osteoblasts was significantly elevated in the bone defect site compared to control intact bone (Figure S1A,B). The elevated fluorescent signal at the nasal area results from a continuous eruption of mouse incisors that requires bone remodelling (Figure S1A). Although anti‐TACE‐mAb did not affect the level of bone deposition, locally injected sSema4D did suppress the mineral deposition (Figure S1A,B). As determined by sSema4D ELISA, the level of sSema4D in the calvaria tissue homogenate was significantly higher in the bone defect site than control bone. However, such elevated sSema4D in the bone defect site was suppressed by systemic anti‐TACE‐mAb administration which was, as noted, regained by local injection of recombinant sSema4D (Figure S1C). The expressions of osteogenic gene markers, *OCN* and *RUNX2* (RUNX Family Transcription Factor 2), were upregulated in the defect site by administration with anti‐TACE‐mAb which was, again, abolished by the additional local injection of recombinant sSema4D (Figure S1D). These results indicated the possible roles of TACE in generating sSema4D, leading to its downregulation of osteoblastogenesis at the critical‐sized bone defect. However, as noted above, anti‐TACE‐mAb had no effect on mineral deposition at the bone defect site. One plausible explanation could be diminished generation of local sSema4D, thus failing to induce sufficient angiogenesis otherwise required to supply nutrients, calcium and phosphates to osteoblasts. A strong body of evidence supports that a timely coordinated angiogenesis is a crucial event for the successful bone repair^17^ and that multifactorial Sema4D possesses angiogenic activity.^18^

We have previously reported that TACE produced by lymphocytes in periodontitis sheds membrane‐bound RANKL to generate sRANKL which, in turn, promotes osteoclastogenesis.^15^ In the present study, we took a step further to prove that TACE has an additional role in the bone remodelling process that of generating sSema4D to inhibit osteoblastogenesis. In conclusion, our study demonstrated that (1) sSema4D was produced by RANKL‐primed osteoclast precursors during the early stage of differentiation when osteoclast cell–cell fusion has not yet begun, (2) Sema4D, TACE and MT1‐MMP were all expressed on the surface of RANKL‐primed osteoclast precursors, but colocalization was only found between Sema4D and TACE, not between Sema4D and MT1‐MMP and (3) TACE‐mediated shedding of Sema4D expressed on the osteoclast precursors generates functionally active sSema4D that can suppress osteoblastogenesis.

## AUTHOR CONTRIBUTIONS

**Takenobu Ishii:** Conceptualization (lead); data curation (lead); formal analysis (lead); investigation (lead); methodology (lead); supervision (lead); validation (lead); writing – original draft (lead); writing – review and editing (lead). **Montserrat Ruiz Torruella:** Conceptualization (supporting); data curation (supporting); formal analysis (supporting); investigation (supporting); methodology (supporting); validation (supporting); writing – review and editing (supporting). **Jae Young Kim:** Data curation (supporting); formal analysis (supporting); investigation (supporting); methodology (supporting); validation (supporting). **Hiroyuki Kanzaki:** Data curation (supporting); formal analysis (supporting); investigation (supporting); methodology (supporting); validation (supporting). **Abdullah Albassam:** Data curation (supporting); formal analysis (supporting); funding acquisition (supporting); investigation (supporting); validation (supporting). **Wichaya Wisitrasameewong:** Data curation (supporting); formal analysis (supporting); funding acquisition (supporting); investigation (supporting); validation (supporting). **Satoru Shindo:** Data curation (supporting); formal analysis (supporting); investigation (supporting); validation (supporting); writing – review and editing (supporting). **Roodelyne Pierrelus:** Data curation (supporting); formal analysis (supporting); funding acquisition (supporting); investigation (supporting); validation (supporting). **Alireza Heidari:** Data curation (supporting); formal analysis (supporting); investigation (supporting); validation (supporting). **Umadevi Kandalam:** Data curation (supporting); formal analysis (supporting); investigation (supporting); validation (supporting). **Shin Nakamura:** Data curation (supporting); formal analysis (supporting); investigation (supporting); validation (supporting). **Alexandru Movila:** Data curation (supporting); formal analysis (supporting); investigation (supporting); validation (supporting); writing – review and editing (supporting). **Dmitriy Minond:** Data curation (supporting); formal analysis (supporting); investigation (supporting); validation (supporting). **Toshihisa Kawai:** Conceptualization (lead); funding acquisition (lead); methodology (lead); project administration (lead); supervision (lead); writing – original draft (lead); writing – review and editing (lead).

## CONFLICT OF INTEREST

The authors declare no conflicts of interest associated with this study.

## DATA AVAILABILTY STATEMENT

The data that support the findings of this study are available from the corresponding authors upon reasonable request.

## Supporting information


Figure S1
