# Supplementary material for: Soluble Sema4D cleaved from osteoclast precursors by TACE suppresses osteoblastogenesis
Source: J Cell Mol Med. 2023 May 11;27(12):1750–6. doi: 10.1111/jcmm.17416 (PMC10273054; doi:10.1111/jcmm.17416)

Supplement Figure

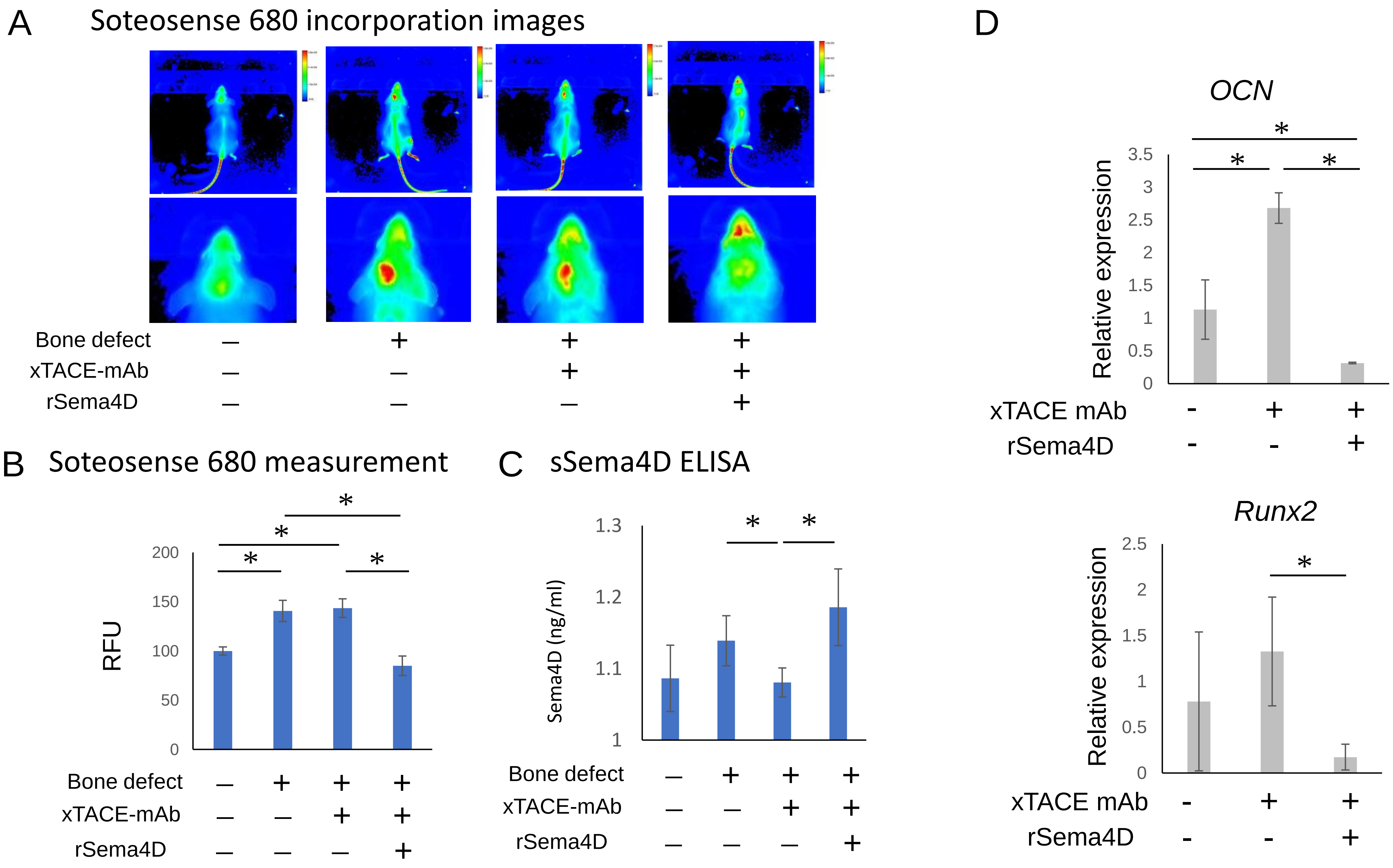

Supplement: Supplementary file 1 — Figure S1 [file JCMM-27-1750-s001.pdf]
